# Real-Time Estimation of Anaerobic Threshold during Exercise Using Electrocardiogram in Heart Failure Patients

**DOI:** 10.3390/jcm12165225

**Published:** 2023-08-11

**Authors:** Toshinobu Ryuzaki, Yasuyuki Shiraishi, Kotaro Miura, Hidehiko Ikura, Yuta Seki, Koichiro Azuma, Kazuki Sato, Keiichi Fukuda, Yoshinori Katsumata

**Affiliations:** 1 Department of Cardiology, Keio University School of Medicine, Tokyo 160-8582, Japan; ryuzaki@keio.jp (T.R.); osapisland@gmail.com (K.M.); ikurahidehiko@yahoo.co.jp (H.I.); y.seki.1028@gmail.com (Y.S.); kfukuda@a2.keio.jp (K.F.); goodcentury21@gmail.com (Y.K.); 2 Institute for Integrated Sports Medicine, Keio University School of Medicine, Tokyo 160-8582, Japan; azumakx@z6.keio.jp (K.A.); kazuki3005@gmail.com (K.S.)

**Keywords:** heart rate variability, ventilatory threshold, cardiopulmonary exercise testing

## Abstract

Exercise therapy at the aerobic level is highly recommended to improve clinical outcomes in patients with heart failure, in which cardiopulmonary exercise testing (CPX) is required to determine anaerobic thresholds (ATs) but is not available everywhere. This study aimed to validate a method to estimate the AT using heart rate variability (HRV) analysis from electrocardiography data in patients with heart failure. Between 2014 and 2019, 67 patients with symptomatic heart failure underwent CPXs in a single university hospital. During the CPX, RR intervals was measured continuously and the HRV threshold (HRVT), defined as the inflection point of <5 ms^2^ of a high-frequency component (HFC) using the power spectrum analysis, was determined. Patients were divided into two groups according to the mean HFC at rest (high-HFC group, *n* = 34 and low-HFC group, *n* = 33). The high-HFC group showed good correlation between the VO_2_ at AT and HRVT (r = 0.63, *p* < 0.001) and strong agreement (mean difference, −0.38 mL/kg, *p* = 0.571). The low-HFC group also showed modest correlation (r = 0.41, *p* = 0.017) but poor agreement (mean differences, 3.75 mL/kg, *p* < 0.001). In conclusion, the HRVT obtained from electrocardiography may be a useful indicator for estimating AT in patients with heart failure.

## 1. Introduction

Exercise therapy is encouraged to improve the functional capacity and health-related quality of life of patients with cardiovascular diseases and to further reduce morbidity and mortality in heart failure patients with left ventricular systolic dysfunction [1], yet its clinical usefulness remains a challenge due to low implementation rates [2,3]; only 7.3% of heart failure patients received an outpatient cardiac rehabilitation program, including exercise therapy in Japan [2]. One barrier to expanding the use of exercise therapy is that healthcare systems are often unwilling to refer patients at risk, such as heart failure, outside the hospital unless a quality and safety exercise program is ensured and controlled. Aerobic-level exercise, clinically defined as anaerobic threshold (AT), safely improves patient outcomes in heart failure [4], while determining AT requires undergoing a cardiopulmonary exercise test (CPX) with a gas analyzer and expertise.

Heart rate variability (HRV) analysis is widely used to assess the cardiac autonomic nervous system, and HRV-rerated indices are associated with mortality and morbidity in patients with myocardial infarction and heart failure [5,6]. In addition, HRV is known to be highly correlated with the AT in healthy volunteers and patients with myocardial infarction and has the potential to predict AT [3,4]. However, information on the association with HRV and AT in heart failure patients who have impaired cardiac autonomic nervous system and often received beta-blockers that can influence an autonomic nervous system is limited. Therefore, this study aimed to estimate AT using a real-time HRV analysis during exercise in heart failure patients without gas analysis. In this context, a number of generic wearable devices capable of measuring HRV integrated with electrocardiogram monitoring are now available and may safely provide aerobic exercise programs based on real-time HRV assessment.

## 2. Materials and Methods

### 2.1. Study Sample

**Figure 1:**
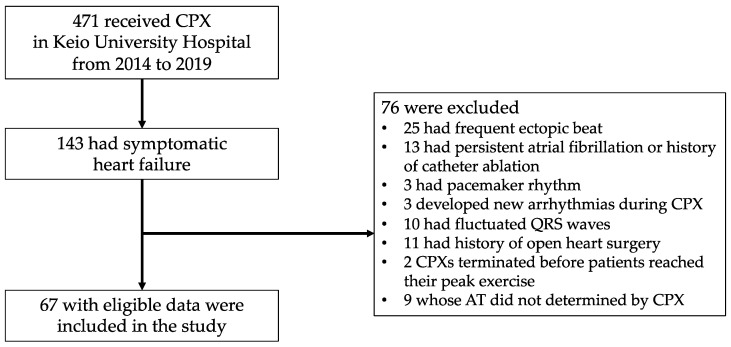
Patients flow chart. CPX, cardiopulmonary exercise test.

During the study period (from 2014 to 2019), 471 patients underwent CPX in Keio University Hospital. Then, 143 patients with symptomatic heart failure were enrolled in this study after excluding 328 patients who had myocardial infarction or atrial fibrillation or underwent CPXs due to the assessment of shortness of breath. Furthermore, seventy-six patients were excluded from this study because of frequent ectopic beats (*n* = 25), concomitant atrial fibrillation or history of catheter ablation for atrial fibrillation (*n* = 13), pacemaker rhythm (*n* = 3), onset of new arrythmia during exercise (*n* = 3), fluctuated QRS wave (*n* = 10), history of open-heart surgery (*n* = 11), and respiratory quotient <1.05 in symptom-limited peak exercise (*n* = 2). We also excluded nine patients whose AT was not determined by CPX. Finally, 67 patients were included in the present study (Figure 1).

### 2.2. Exercise Test Protocol

An incremental symptom-limited exercise test was performed with an electro-magnetically braked ergometer (Strength Ergo 8, Fukuda Denshi, Tokyo, Japan) according to the ramp protocol. The test consisted of a 2 min resting period, followed by 2 min of warm-up at an ergometer setting of 0 W (60 rpm), followed by testing with a 1 W increase in exercise load every 4–6 s (10–15 W/min) depending on the predicted maximum exercise capacity and in such a way that maximal effort was attained within 8 to 15 min. During the test, heart rate (HR), blood pressure, oxygen saturation, and electrocardiogram were recorded and monitored continuously in all subjects.

During exercise, oxygen consumption (VO_2_), carbon dioxide production (VCO_2_), and minute ventilation (VE) were measured using the 10-s average with a metabolic cart (AE-302S; MINATO, Tokyo, Japan). Peak VO_2_ was calculated as the average VO_2_ during the last 30 s of exercise. The anaerobic threshold (AT) point was determined using the V-slope method in addition to the following conventional criteria: VE/VO_2_ increases after registering as flat or decreasing, whereas VE/VCO_2_ remains constant or decreases [7]. First, 2 of 3 experienced researchers independently and randomly evaluated the AT of each subject through the above methods. Second, if the VO_2_ values determined by the independent researchers were within 3%, then the VO_2_ values for the 2 investigators were averaged. Third, if the VO_2_ values determined by the independent evaluators were not within 3% of one another, a third researcher then independently determined VO_2_. The third VO_2_ value was then compared with those of the initial investigators. If the adjudicated VO_2_ value was within 3% of either of the initial investigators, then 2 VO_2_ values were averaged [3]. The VE vs. VCO_2_ slope was calculated from the start of incremental exercise to the respiratory compensation point using least squares linear regression analysis.

### 2.3. HRV Measurement

**Figure 2:**
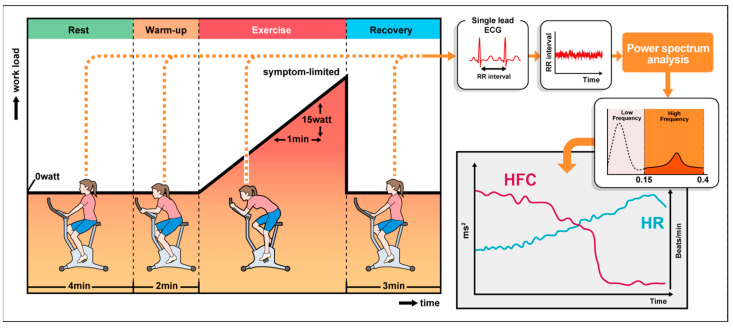
Cardiopulmonary exercise testing protocol and the methods of power spectrum analysis of heart rate variability. ECG, electrocardiogram; HR, heart rate; HFC, high-frequency component.

The ECG data were stored with a sampling rate of 1000 Hz by LRR-03^®^ (Crosswell, Yokohama, Japan). Reflex Meijin^®^ (Crosswell, Yokohama, Japan) was used to automatically measure the RR intervals (beat to beat fluctuation of heart rate) of the subjects at 1000 Hz during CPX. The data of RR intervals were instantaneously stored for real-time analyses. Based on the data of RR intervals, power spectral densities were computed continuously by the maximum entropy method analyzing the RR intervals for 30 s using the Reflex Meijin^®^. Power spectral analysis of the HRV mainly describes the high-frequency component (HFC; 0.15–0.40 Hz frequency band) and low-frequency component (LFC; 0.04–0.15 Hz frequency band) [8]. The HFC reflects the cardiac parasympathetic nervous tone [9,10]. After storing the data of RR intervals for the first 30 s at rest, the power continued to be quantified in an HFC and LFC whilst updating the data every heartbeat. With the continuous analysis of every heartbeat, the power spectrum was projected on the screen without delay during the CPX (Figure 2). Based on our previous study, the inflection point that an HFC disappears with <5 ms^2^ was defined as the HRV threshold (HRVT) [3].

### 2.4. Statistical Analysis

The results are represented as the median with an interquartile range for continuous variables and as percentages for categorical variables, as appropriate. The null hypothesis indicated that the mean difference between the AT-VO_2_ and HRVT-VO_2_ was equal to zero. The relationships among the studied methods of the AT-VO_2_ and HRVT-VO_2_ were investigated by the Pearson correlation coefficient test. In addition, the Bland and Altman technique was applied to verify the similarities among the different methods (AT and HRVT). In addition, we divided the patients into two groups based on resting HFC as differences in correlation coefficients are expected depending on the HFC value [3]. All probability values were 2-tailed, and *p* values of <0.05 were considered to be statistically significant. All statistical analyses were performed with SPSS version 23.0 software (SPSS Inc., Chicago, IL, USA).

## 3. Results

### 3.1. Patient’s Backgroud 

The patients were predominantly male (69%), with a median age and left ventricular ejection fraction (LVEF) of 59 (46–68) years and 37.7% (30.8–47.7), respectively. Sixty-three (94%) patients were taking beta-blockers. 

Patients were divided into two groups according to the mean value (73.9 ± 147.8 ms^2^) of HFC at rest (high-HFC group, *n* = 34 and low-HFC group, *n* = 33). Compared with patients in the high-HFC group, those in the low-HFC group had a higher estimated pulmonary arterial pressure on echocardiography (*p* < 0.05) (Table 1). B-type natriuretic peptide (BNP) levels in the low-HFC group were higher than that in the high-HFC group, although the difference was not statistically significant (*p* = 0.054). No significant differences were found in a dose of beta-blockers, LVEF, history of diabetes mellitus, and underlying etiologies of heart failure (i.e., ischemic or non-ischemic). The angiotensin receptor-neprilysin inhibitor was not used in both groups because it was an unapproved drug material in Japan during this study period. In regard to CPX parameters, the low-HFC group had a significantly higher HR and VE-VCO_2_ slope than the high-HFC group, although there was no between-difference in peak HR reserve.

### 3.2. HRV Analysis

**Figure 3:**
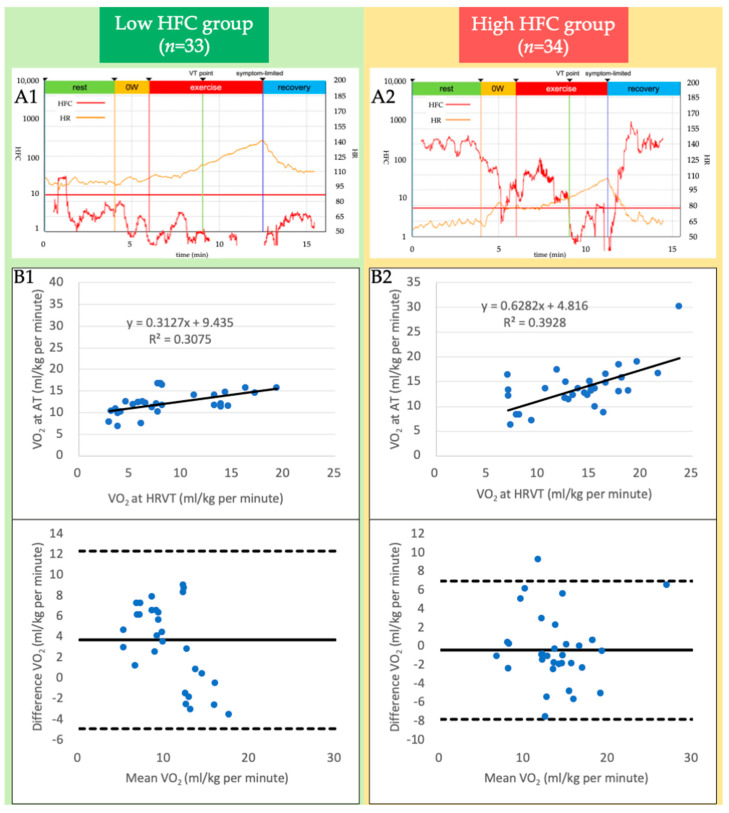
(**A1**,**A2**) Quantitative imaging of the power spectrum of heart rate variability (HRV) during incremental exercise. A representative graph of the high-frequency component (HFC) (red) and heart rate (HR; orange) with an RAMP (15 W/min) protocol ergometer is shown in the low-HFC (**A1**) and high-HFC groups (**A2**), respectively. (**B1**,**B2**) Validity testing of the oxygen uptake (VO_2_) at the HRV threshold (HRVT) and anaerobic threshold (AT) in the low-HFC (**B1**) and high-HFC groups (**B2**). The graph in the upper panel shows the relationship between each VO_2_ at the HRVT and AT, and that in the lower panel shows the Bland-Altman plots, which indicate the respective differences between each VO_2_ at the HRVT and AT (y-axis) for each individual against the mean of the VO_2_ at the HRVT and AT (x-axis). The dark line in the Bland-Altman plot represent a ± 1.96 standard deviation.

The power spectrum of the HRV during the CPX was completely visualized in both groups. The correlation coefficient between the VO_2_ at AT and HRVT was modest (r = 0.52, *p* < 0.001) in the entire cohort. The Bland–Altman plot shows a mild agreement (mean difference, 1.71 mL/kg, *p* = 0.014). The HFC component decreased drastically with an HFC peak after starting the exercise and disappeared after the AT in the high-HFC group, while the low-HFC group showed a steady-state or a vacillated pattern of the HFC component (Figure 3A). The high-HFC group showed a good correlation between the VO_2_ at AT and HRVT (r = 0.63, *p* < 0.001). The Bland–Altman plot also described a strong agreement (mean difference, −0.38 mL/kg, *p* = 0.571) (Figure 3B). On the contrary, the low-HFC group showed a modest correlation (r = 0.41, *p* = 0.017) but a poor agreement (mean differences, 3.75 mL/kg, *p* < 0.001). A sensitivity analysis that excluded six patients receiving antiarrhythmic agents that affect HR (e.g., digoxin, amiodaron, etc.) yielded similar results to the main analysis. 

## 4. Discussion

This study demonstrated that the HRVT using our real-time HRV assessment significantly correlated with the AT in patients with heart failure. Gas analysis parameters may oscillate, and exercise load may be completed in a very short period of time among patients with heart failure, leading to difficulty with AT estimation. Therefore, real-time HRV assessment can be a useful tool to assist in the diagnosis of AT in such cases. There are now no clear recommendations to a specific exercise modality, such as continuous aerobic exercise training vs. high-intensity interval training, in patients with heart failure [12]; however, the higher the exercise intensity, the higher the risk and the greater the need for adjustments to ensure safety and efficacy. To promote exercise therapy outside of hospital with reliable monitoring systems, continuous aerobic exercise training using real-time HRV analysis may be safe and effective in patients with heart failure and relatively mild to moderate severity.

The accurate identification of HRs close to AT is actually useful in promoting effective rehabilitation programs. Magrì et al. [13] reported that the range 75–80% of maximal estimated HR was the most accurate in identifying the HR at the AT among heart failure patients. In contrast, chronotropic incompetence (defined as the peak HR reserve < 70%) was prevalent in one-third of heart failure patients and was significantly associated with a risk of cardiovascular mortality [14]. Chronotropic incompetence is also known to be associated with advanced stages of heart failure severity, though no apparent relationship was found between chronotropic incompetence and HFC groups in our study. Taken together, both HR- and HRV-based estimation of AT without CPX remains a challenge in advanced heart failure patients.

We also found that patients in the low-HFC group showed higher severity of heart failure grading with a higher HR, pulmonary artery pressure, and VE-VCO_2_ slope. The decrease in the HFC of the HRV has been known to be associated with advanced heart failure [15], which is compatible with our findings. In addition, the change in HFC during exercise in the low-HFC group was not constant and uniform, thereby indicating the limited association with HRVT and AT. We previously reported that a very small number of patients with myocardial infarction but not heart failure had a much lower HFC, in which we were unable to estimate their AT using the same method of this study [3]. The fact may suggest that these patients had a severely impaired cardiac autonomic nervous system and/or activated their sympathetic nervous systems even at rest.

This study has several limitations. Firstly, it was conducted at a single university hospital on highly selected patients; therefore, the results may not be generalizable to the heart failure patient population of other countries. Secondly, as this study was a cross-sectional retrospective study, data on patient’s prognosis or health-related quality of life were not assessed. In addition, one of the central roles of CPX is the prognostication of patients with heart failure, and thus the clinical usefulness and significance of our method would not be as extensive as CPX. Finally, approximately 30% of patients with heart failure complicate atrial fibrillation, but the HRV analysis is applicable exclusively to patients with sinus rhythm but not to atrial fibrillation. However, the accuracy and validity of AT estimation in patients with atrial fibrillation is also known to be problematic [16]. The development of AT-guided exercise therapy specifically tailored for these patients needs to address the challenges associated with AT assessment. Although some inherent challenges, such as pacemaker rhythm, limit the universal applicability of our method, in principle it could be applied to at least more than half of heart failure patients.

## 5. Conclusions

Among patients with symptomatic heart failure, there is a potential for real-time HRV assessment using a single-lead electrocardiogram to estimate AT without any gas analyzer and expertise. However, it is important to approach these findings with caution as they are based on limited results from a small number of patients and currently remain in the realm of hypotheses. To establish the clinical utility and validity of this approach, further validation studies are required in a larger cohort of heart failure patients. 

## Figures and Tables

**Table 1:** Patient demographics, echocardiography, and cardiopulmonary exercise testing data.

	Low-HFC Group (*n* = 33)	High-HFC Group (*n* = 34)
Baseline characteristics		
Age, years	60 (49–64)	55 (40–68)
Male, *n* (%)	24 (73)	22 (65)
Body mass index, kg/m^2^	22.9 (21.2–26.1)	22.9 (19.8–25.2)
Diabetes mellitus, *n* (%)	9 (31)	7 (18)
Ischemic etiology, *n* (%)	4 (12)	5 (15)
Non-ischemic etiology, *n* (%)	29 (88)	29 (85)
Beta-blocker, *n* (%)	31 (94)	32 (94)
Dose of β-blocker, mg	5.0 (2.5–10.0)	10.0 (4.7–20.0)
ACEI or ARB, *n* (%)	27 (82)	29 (85)
MRA, *n* (%)	23 (70)	21 (62)
Laboratory data		
eGFR, mL/min/1.73 m^2^	61 (41–77)	59 (39–75)
Hemoglobin, g/dL	12.6 (11.6–13.9)	13.4 (12.4–14.6)
HbA1c, %	5.9 (5.5–6.3)	5.8 (5.5–6.5)
BNP, pg/mL	93.7 (28.5–381.8)	64.2 (20.8–167.5)
Echocardiography data		
LVEF, %	36.9 (27.4–43.9)	37.1 (32.5–50.0)
LVDd, mm	60 (50.5–65.0)	58 (51–62)
LVDs, mm	48 (38.5–56.0)	48 (34.8–52.5)
PASP, mm Hg	29 (22–41)	25 (20–31) *
CPX data		
At rest		
HR, bpm	81 (70–90)	74 (66–79) *
Systolic BP, mmHg	119 (102–130)	117 (103–128)
VO_2_, mL/kg per min	3.8 (3.4–4.3)	3.7 (3.4–4.2)
HFC, ms^2^	13.7 (7.0–19.7)	94.3 (43.2–120) *
During warm-up		
HR, bpm	88 (75–97)	81 (75–85) *
Systolic BP, mmHg	127 (112–141)	128 (117–144)
VO_2_, mL/kg per min	6.7 (6.0–7.9)	6.3 (5.8–7.6)
At anaerobic threshold		
HR, bpm	102 (92–114)	100 (89–109)
Systolic BP, mmHg	138 (119–153)	138 (119–153)
VO_2_, mL/kg per min	12.0 (11.0–14.9)	13.2 (11.6–15.3)
VO_2_ percentage of predicted peak VO_2_, %	46 (37–59)	46 (37–64)
RQ	0.90 (0.87–0.97)	0.91 (0.82–0.95)
At peak exercise		
HR, bpm	140 (115–147)	134 (117–149)
Systolic BP, mmHg	160 (140–178)	155 (130–178)
VO_2_, ml/kg per minute	19.2 (14.6–26.8)	19.8 (16.6–26.6)
VO_2_ percentage of predicted peak VO_2_, %	71 (59–91)	77 (57–94)
RQ	1.15 (1.09–1.22)	1.17 (1.09–1.24)
Peak HR reserve, % ^#^	65 (48–80)	62 (50–71)
VE-VCO_2_ slope	30.2 (26.6–35.3)	29.2 (26.2–30.8) *

The values are represented as the median with an interquartile or numbers (percentages). ACEI indicates angiotensin-converting enzyme inhibitor; ARB, angiotensin receptor blocker; MRA, mineral corticoid receptor antagonist; eGFR, estimated glomerular filtration rate; HbA1c, hemoglobin A1c; BNP, B-type natriuretic peptide; LVEF, left ventricular ejection fraction; LVDd, left ventricular end-diastolic diameter; LVD, left ventricular end-systolic diameter; PASP, estimated pulmonary arterial systolic pressure on echocardiography; HR, heart rate; BP, blood pressure; VO_2_, oxygen uptake; HFC, high-frequency component; RQ, respiratory quotient; VE-VCO_2_, ventilation–carbon dioxide production. * *p* < 0.05. Equivalent to 10 mg of carvedilol and 2.5 mg of bisoprolol. # Peak HR reserve was calculated as the observed HR divided by the predicted maximum HR [11].

## Data Availability

The data underlying this article will be shared on reasonable request to the corresponding author.

## References

[1] Yancy C.W., Jessup M., Bozkurt B., Butler J., Casey D.E., Drazner M.H., Fonarow G.C., Geraci S.A., Horwich T., Januzzi J.L. (2013). 2013 ACCF/AHA guideline for the management of heart failure: A report of the American College of Cardiology Foundation/American Heart Association Task Force on Practice Guidelines. J. Am. Coll. Cardiol..

[2] Kamiya K., Yamamoto T., Tsuchihashi-Makaya M., Ikegame T., Takahashi T., Sato Y., Kotooka N., Saito Y., Tsutsui H., Miyata H. (2019). Nationwide Survey of Multidisciplinary Care and Cardiac Rehabilitation for Patients with Heart Failure in Japan—An Analysis of the AMED-CHF Study. Circ. J..

[3] Shiraishi Y., Katsumata Y., Sadahiro T., Azuma K., Akita K., Isobe S., Yashima F., Miyamoto K., Nishiyama T., Tamura Y. (2018). Real-Time Analysis of the Heart Rate Variability During Incremental Exercise for the Detection of the Ventilatory Threshold. J. Am. Heart Assoc..

[4] Cottin F., Medigue C., Lepretre P.M., Papelier Y., Koralsztein J.P., Billat V. (2004). Heart rate variability during exercise performed below and above ventilatory threshold. Med. Sci. Sports Exerc..

[5] La Rovere M.T., Bigger J.T., Marcus F.I., Mortara A., Schwartz P.J. (1998). Baroreflex sensitivity and heart-rate variability in prediction of total cardiac mortality after myocardial infarction. ATRAMI (Autonomic Tone and Reflexes After Myocardial Infarction) Investigators. Lancet.

[6] La Rovere M.T., Pinna G.D., Maestri R., Mortara A., Capomolla S., Febo O., Ferrari R., Franchini M., Gnemmi M., Opasich C. (2003). Short-Term Heart Rate Variability Strongly Predicts Sudden Cardiac Death in Chronic Heart Failure Patients. Circulation.

[7] Gaskill S.E., Ruby B.C., Walker A.J., Sanchez O.A., Serfass R.C., Leon A.S. (2001). Validity and reliability of combining three methods to determine ventilatory threshold. Med. Sci. Sports Exerc..

[8] Niewiadomski W., Gasiorowska A., Krauss B., Mroz A., Cybulski G. (2007). Suppression of heart rate variability after supramaximal exertion. Clin. Physiol. Funct. Imaging.

[9] Pomeranz B., Macaulay R.J., Caudill M.A., Kutz I., Adam D., Gordon D.A.V.I.D., Kilborn K.M., Barger A.C., Shannon D.C., Cohen R.J. (1985). Assessment of autonomic function in humans by heart rate spectral analysis. Am. J. Physiol..

[10] Pagani M., Lombardi F., Guzzetti S., Rimoldi O., Furlan R.A., Pizzinelli P., Sandrone G., Malfatto G., Dell’Orto S., Piccaluga E. (1986). Power spectral analysis of heart rate and arterial pressure variabilities as a marker of sympatho-vagal interaction in man and conscious dog. Circ. Res..

[11] Tanaka H., Monahan K.D., Seals D.R. (2001). Age-predicted maximal heart rate revisited. J. Am. Coll. Cardiol..

[12] Mueller S., Winzer E.B., Duvinage A., Gevaert A.B., Edelmann F., Haller B., Pieske-Kraigher E., Beckers P., Bobenko A., Hommel J. (2021). Effect of High-Intensity Interval Training, Moderate Continuous Training, or Guideline-Based Physical Activity Advice on Peak Oxygen Consumption in Patients with Heart Failure with Preserved Ejection Fraction: A Randomized Clinical Trial. JAMA.

[13] Magrì D., Piepoli M., Gallo G., Corrà U., Metra M., Paolillo S., Filardi P.P., Maruotti A., Salvioni E., Mapelli M. (2022). Old and new equations for maximal heart rate prediction in patients with heart failure and reduced ejection fraction on beta-blockers treatment: Results from the MECKI score data set. Eur. J. Prev. Cardiol..

[14] Magrì D., Corrà U., Di Lenarda A., Cattadori G., Maruotti A., Iorio A., Mezzani A., Giannuzzi P., Mantegazza V., Gondoni E. (2014). Cardiovascular mortality and chronotropic incompetence in systolic heart failure: The importance of a reappraisal of current cut-off criteria. Eur. J. Heart Fail..

[15] Casolo G.C., Stroder P., Sulla A., Chelucci A., Freni A., Zerauschek M. (1995). Heart rate variability and functional severity of congestive heart failure secondary to coronary artery disease. Eur. Heart J..

[16] Agostoni P., Emdin M., Corrà U., Veglia F., Magrì D., Tedesco C.C., Berton E., Passino C., Bertella E., Re F. (2008). Permanent atrial fibrillation affects exercise capacity in chronic heart failure patients. Eur. Heart J..

